# Scrutinizing the Nutritional Aspects of Asian Mushrooms, Its Commercialization and Scope for Value-Added Products

**DOI:** 10.3390/nu14183700

**Published:** 2022-09-07

**Authors:** Judy Gopal, Iyyakkannu Sivanesan, Manikandan Muthu, Jae-Wook Oh

**Affiliations:** 1 Department of Research and Innovation, Saveetha School of Engineering, Saveetha Institute of Medical and Technical Sciences (SIMATS), Thandalam, Chennai 602105, India; 2 Department of Bioresources and Food Science, Institute of Natural Science and Agriculture, Konkuk University, 1 Hwayang-dong, Gwangjin-gu, Seoul 05029, Korea; 3 Department of Stem Cell and Regenerative Biotechnology, Konkuk University, 1 Hwayang-dong, Gwangjin-gu, Seoul 05029, Korea

**Keywords:** mushrooms, nutritional, food, commercial products, nutraceutical

## Abstract

Mushrooms are the gifts of the non-green revolution; they are not limited by land demand or specific growth requirements. Nearly 14,000 species of mushrooms are on record thus far; of these, only 2200 species are deemed edible. Only 650 species from this list have been cultivated and consumed. Farmed on waste, mushrooms are rich reservoirs of proteins, polysaccharides, metabolites, minerals and vitamins. In the following review, various edible mushrooms have been listed and their nutritional aspects and their associated contributions have been discussed. Furthermore, the commercial mushroom-based products that are on the market have been surveyed. The challenges facing the use of mushroom and mushroom products as foods, functional foods and nutraceuticals have been presented. The need to seek options to troubleshoot the current limitations has also been discussed.

## 1. Introduction

Mushrooms, described as the “elixir of life” in Chinese medicine, are classified superfoods with impressive nutritional benefits. They are packed with immunity boosters and nutrients. They have been an integral part of traditional medicine for thousands of years, dating back to the time of the ancient Egyptians. The developing nations are currently struggling to meet the food demand of the increasing population, owing to limited land resources and climate change. Widespread malnutrition and associated diseases are also prevalent among economically poor populations. There is a compelling need to constantly look out for cheaper alternative nutritional sources for our large population. Mushroom farming, also referred to as the non-green revolution, is among one of the few choices to meet the existing nutritional demand. The main advantage is the fact that mushroom grow on wastes without requiring additional land, in addition its exceptional nutritional and medicinal properties [1].

**Figure 1:**
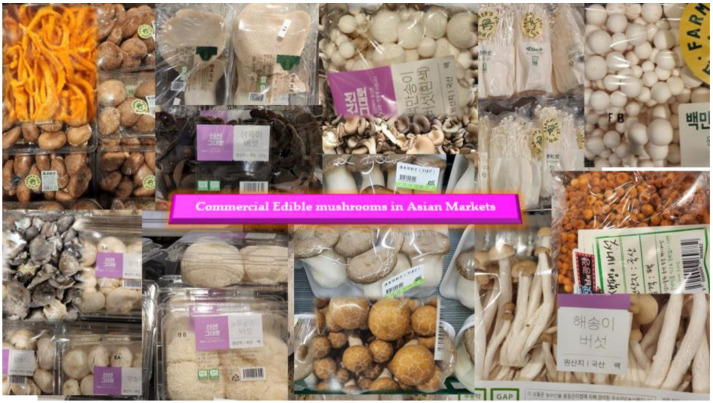
Overview of the various edible mushroom varieties in the Korean market.

Mushrooms are universally accepted for their texture and flavor, but more so for their chemical, nutritional [2] and functional attributes [3]. Edible and wild mushrooms are available, and are globally appreciated for their high mineral and water content, along with the fact that they are rich reservoirs of hydrocolloids, such as proteins, fibers, and carbohydrates. Mushrooms are low on fats and are ideal for inclusion into low caloric diets [4,5,6]. They are highly nutritive, with nutritional scales on par with meat, eggs and milk, as they have an almost identical amino acid composition comparable to animal proteins [2,7]. Mushrooms are considered a unique delicacy, and well known for their specific unami aroma and texture. Figure 1 presents a bird’s eye view of the various mushroom varieties in the Korean fresh market.

**Figure 2:**
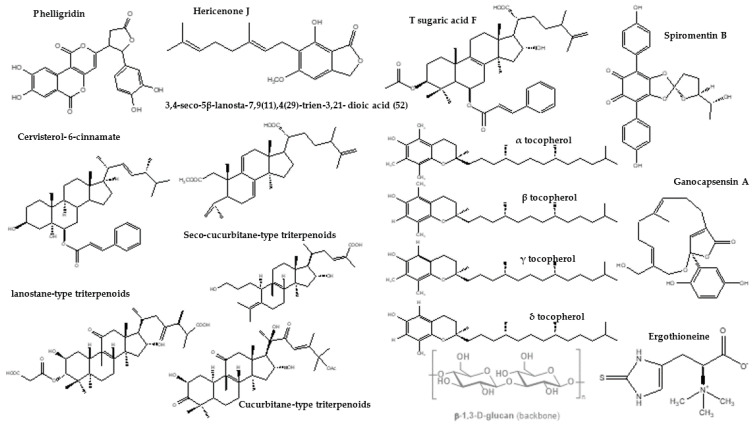
Chemical structures of a few representative mushroom-based nutraceuticals.

With wheat, rice and maize being the staple food of a vast majority of the global population, there is a marked deficit of protein in our diets. Supplementation of mushrooms bridges the protein gap and improves nutrition and wellbeing of socially and economically backward communities. In the past, mushrooms were categorized as expensive/exotic vegetable types, and consumed only by the elite. Nowadays, thanks to the increasing awareness, these are made available through local mushroom farmers to the general public. Mushrooms hold holistic nutrition, suitable for all age groups. The nutritional value of mushrooms is strongly influenced with respect to the species, stage of development and environmental conditions. Mushrooms contain surplus protein, dietary fiber, vitamins and minerals, as well as starches, pentoses, hexoses, disaccharides, amino sugars, sugar alcohols and sugar acids. The carbohydrate content of mushrooms ranges from 26 to 82%. Their crude fiber is made up of partially digestible polysaccharides and chitin. Mushrooms commonly have low lipid levels, yet they contain polyunsaturated fatty acids in large proportions. Thus, they have low calorific yields and no cholesterol. They also contain ergosterol (Vit-D precursor), proving to be highly advantageous [1,8,9,10,11]. Figure 2 shows a few representative chemical structures of bioactive moieties in edible mushrooms.

Shiitake is a famous mushroom variety that contains eight essential amino acids, as well as linoleic acid. Shiitake when consumed can enhance satiety, which is ideal for weight loss. In addition, they also are abundant in phytonutrients, which are responsible for health and immune function. Porcini mushrooms add unique flavor to broths, and are incorporated into bread or pasta flour. These mushrooms are rich in antioxidants, beta-carotene, ascorbic acid and lycopene and fiber and plant-based protein. Chaga has an earthy flavor, and it contains vanillin; it is the best food when it comes to oxygen radical absorbent capacity, implying its potential to become a rich source of antioxidants and help in disease prevention. Chaga mushrooms reduce inflammation and improve physical endurance. Lion’s mane has brain-boosting properties, fighting Alzheimer’s. It also is ideal fort he heart and digestive health because of its anti-inflammatory potential. Reishi mushrooms have been valued by Chinese populations for thousands of years. They go by the nickname “king of mushrooms” and prompt the body to tackle negative effects of stress. They are a rich reservoir of antioxidants and health-promoting polysaccharides. Reishi mushrooms balance hormones, promote heart health and stabilize blood sugar levels. Cordyceps are valuable resources for holistic medicine and are termed superstar supplements that accelerate athletic performance, reduce inflammation and promote cardiovascular health [12]. These muchrooms are rich in antioxidants that combat diseases, boost immune function and slow aging. Lion’s mane mushrooms reduce the impact of neurodegenerative diseases, chaga stimulates spleen lymphocytes that regulate the immune system and boost the body’s ability to fight off invading viruses and bacteria. Traditionally, mushrooms have been used to reduce stress and anxiety. Reishi is known for its antidepressant potential and can ease stress-induced anxiety. Shiitake mushrooms have vitamin B and support adrenal function and can turn the nutrients to useable energy. The polysaccharides in mushrooms enable their physical endurance. Medicinal mushrooms help deal with diabetes and improve blood glucose levels and insulin sensitivity. Cordyceps could help prevent heart damage and lower LDL cholesterol. Mushrooms can protect the organ and heart by fighting oxidative stress and can slow down aging and optimize overall health [13,14]. Numerous unexplored wild mushrooms are also being unearthed and the nutritional value of these wild mushrooms cannot and should not be overlooked [15].

Apart from their nutritional value, many of the mushroom species have therapeutic properties and are sought after for their medicinal properties [16]. Numerous studies have confirmed that the antioxidant properties of mushrooms operate via their phenolic and flavonoid compounds [17,18,19,20,21]. Mushrooms also help in reducing Parkinson’s disease, Alzheimer’s disease, hypertension, strokes, and cancer, as well as act as antibacterial, antiviral, immune system enhancers and cholesterol-moderating agents [22]. Mushrooms are also known to prevent hypertension, hypercholesterolemia, and cancer, as well as protect the cells from free radical, ageing, associated ailments [23]. According to Zekovic et al. [24], mushrooms contain β-glucans that display antitumour, immune-booster effects. T β-glucans produce hold nomenclatures, such as ganoderan (*Ganoderma lucidum*), grifolan (Grifola fondosa), lentinan (*Lentinus edodes*), pleuran (*Pleurotus ostreatus*), and schizophyllan (*Schizophyllum commune*) [25] and these are well known for their antioxidant, anticancer, anticholesterolaemic, immunomodulating, neuroprotective and antibacterial [26] activities. Furthermore, they are also documented for their role as immunological stimulators in humans and bind to a membrane receptor and induce various biological responses [25,27,28]. Valverde et al. [22] have also reported the presence of phenolics, ascorbic acid, carotenoids, and tocopherols in mushrooms that are responsible for orchestrating a host of bioactivities; in addition to that, they are also able to ameliorate the toxic effects generated through chemo- and radiotherapy. Lau et al. [29] have shown that the protein extracts from *Pleurotus cystidiosus* and *Agaricus bisporus* have high antihypertensive activities, Pleuran from *Pleurotus spp*. exhibits immunity-stimulating effects, blood cholesterol reduction and immunomodulatory and antitumor activities [30] (Agrawal et al. 2010). Li et al. [31] reported that *Pholiota nameko* polysaccharide (PNPS-1) could bring about a significant decrease in very low-density lipoprotein/low-density lipoprotein cholesterol and an increase in high-density lipoprotein cholesterol.

In the following review, the various mushrooms available in the Asian market are reviewed for their nutritional assets. A comprehensive compilation of their nutritional attributes and a brief overview of their commercialized value-added products have been presented and discussed. The lapses in harnessing the full potential of these valuable assets have been presented and the recommendations to overcome these challenges put forth.

## 2. Comprehensive Listing of Edible Mushrooms

It is recorded that, as of now, 14,000 species of mushrooms exist. These species can be classified as edible mushrooms, magic mushrooms and poisonous mushrooms. Magic mushrooms are known to exhibit hallucinogenic properties. Psilocybin, the psychedelic compound in hallucinogenic mushrooms, holds mind-altering effects. These mushrooms grow wild and are classified as a schedule 1 drug in the United States. The group categorized as poisonous mushrooms are also known to exist. These can lead to mild gastrointestinal discomfort to even death. The existence of poisonous mushrooms has resulted in their limited popularity and the widespread use of edible mushrooms, owing to the reason that poisonous mushrooms very closely resemble edible varieties; it is important to correctly identify any wild-harvested mushrooms before adding them to your favorite meal. Technically speaking, a mushroom is termed edible only if it can be eaten without any psychological changes or harm to the body. This group makes up the vast majority of known mushrooms; however, most are too bitter, tough, or slimy to be used in cooking. In addition, it is also a known fact that many mushrooms that are edible are only edible during certain life stages or after being cooked. With this introduction, we give a brief snapshot of the various mushrooms that are available, such as the bay bolete mushroom (*Boletus badius*), black trumpet mushroom (*Craterullus cornocopioides*), button mushroom (*Agaricus bisporus*), Caesar’s mushroom (*Amanita caesarea*), cauliflower mushroom (*Sparassis*), chanterelle mushrooms (*Cantharellus cibarius*), charcoal burner mushroom (*Russula cyanoxantha*), chicken of the woods mushroom (*Laetiporus sulphureus*), common ink cap mushroom (*Coprinopsis atramentaria*), crab brittlegill mushroom (*Russula xerampelina*), cremini mushroom (*Agaricus bisporus*), dryad’s saddle (*Cerioporus squamosus*), enoki mushroom (*Flammulina velutipes*), false morel mushroom (*Verpa bohemica*), field mushroom (*Agaricus campestris*), giant puffball mushroom (*Calvatia gigantea*), green cracking russula (*Russula virescens*), gypsy mushroom (*Cortinarius caperatus*), hedgehog mushroom (*Hydnum umbilicatum*), honey fungus mushroom (*Armillaria ostoyae*), king bolete mushroom (*Boletus edulis*), king oyster mushroom (*Pleurotus eryngii*), oyster mushroom (*Pleurotus ostreatus*), lion’s mane mushroom (*Hericium erinaceus*), maitake mushroom (*Grifola frondosa*), morel mushrooms (Morchella), matsutake mushrooms (*Tricholoma matsutake*), parasol mushroom (*Macrolepiota procera*), portobello mushroom (*Agaricus bisporus*), red pine mushroom (*Lactarius deliciosus*), red-capped scaber stalk (*Leccinum aurantiacum*), reishi mushroom (*Ganoderma lingzhi*), buma shimeji mushrooms (*Hypsizygus tessellatus*), shiitake mushrooms (*Lentinula edodes*), slippery Jack mushrooms (*Suillus luteus*), straw mushrooms (*Volvariella volvacea*), wood blewit (*Clitocybe nuda*), wood ear mushrooms (*Auricularia auricula-judae*), yellow knight mushroom (*Tricholoma equestre*) and reshi/lingzhi mushroom (*Ganoderma lucidum*). Some highly poisonous varieties include death cap (*Amanita phalloides*), which are among the most poisonous of all mushrooms and responsible for the majority of mushroom-related deaths worldwide, *Conocybe filaris*, which contains the same toxins as death caps, autumn skullcap (*Galerina marginata*), also known as the “deadly galerina,” and are among the most poisonous of mushrooms, death angel (*Amanita ocreata*), which can cause severe illness and death if eaten [24] and false morels (*Gyromitra esculenta* and *Gyromitra infula*), which also fall under the category of poisonous mushrooms [32,33,34,35,36].

## 3. An Overview of the Nutritional Value of Mushrooms

Mushrooms contain eighty to ninety percent water, and eight to ten percent fiber. In addition to these, mushrooms are an excellent source of vitamin C and B (folic acid, thiamine, riboflavine and niacin). Potassium, sodium and phosphorous minerals are abundant in fruit bodies of the mushroom and other essential minerals, such as Cu, Zn and Mg in traces [37,38]. It is also reported [39] that mushrooms provide both digestible carbohydrates (i.e., trehalose, mannitol, glycogen, and glucose) and non-digestible carbohydrate (i.e., mannans, chitin, and β-glucan). Aremu et al. [40] calculated the metabolizable energy levels of Ganoderma spp. as 1476.7 kJ/100 g and those in *Hebeloma mesophaeum* as 1513.5 kJ/100 g, indicating that both these mushroom types have energy values that are on par with cereals, in addition to their high protein contents [41,42]; in this way, it can be said that mushrooms are, thus, considered to be richer than most food sources (except meat) [41]. In addition to this, mushrooms also contain several essential amino acids, such as phenylalanine, lysine, isoleucine, leucine, valine, histidine, threonine, methionine, glutamic acids, and aspartic [43]. Glutamic acids and aspartic are the two essential amino acids that give the umami taste that is unique to mushrooms [32,44]. Thus, mushrooms provide a balanced diet ideal for human health [1,2,45,46].

According to Elleuch et al. [47], polysaccharides and dietary fibers are abundant in different parts of mushrooms, especially in the cap, stalk, of *Pleurotus ostreatus* [48]. In another study by Kayode et al. [38], proximate compositions of oyster mushrooms cultured on Gmelina wood waste, when compared with wild species of oyster mushrooms, revealed variable nutrient contents. The oyster mushroom grown on Gmelina wood waste in this case had higher nutrient contents than its wild counterpart [38]. Valverde et al. [22] reported that linoleic (C18:2), oleic (C18:1), and palmitic (C16:0) acids are major fatty acids found in mushrooms [40]. Alexopolous et al. [49] documented that the fairy ring mushroom (*Marasmius oreades*) contains copper, iron, zinc, folic acid, besides the other essential amino acids that play an essential role in human nutrition and dietetics. *Lentinula edodes* has low sodium and low glucose levels, which makes it attractive for diabetics [49]. Oluwafemi et al. [48] reported that phosphorus, magnesium, and potassium were the major minerals in mushrooms. In addition, according to Kalač [50], potassium is most abundant element in edible mushrooms. Magnesium is the second major mineral preceding potassium and sodium is the lowest [50,51]. This trend is, nutritionally, the ideal balance and is naturally available in mushrooms.

Another report has documented the presence of β-carotene (vitamins A); α-tocopherol and γ-tocopherol for vitamin E, ascorbic acid (vitamin C), thiamine (vitamin B1), and riboflavin (vitamin B2); and several dominant and trace minerals in *Pleurotus* sp., *Hygrocybe* sp., *Hygrophorus* sp., *Schizophyllum commune*, and *Polyporus tenuiculus*. *Schizophyllum commune* was identified to possess the highest vitamin A and E levels [37,52]. According to Keegan et al. [53], mushrooms generally lack vitamin D2, but they function as biological precursors to vitamin D2 due to the presence of ergosterol, which is a type of sterol predominantly found in mushrooms. Table 1 shows the nutritional aspects of mushrooms.

Mushrooms have also been used as a nutraceutical, and their vast array of bioactive compounds have been applied as therapeutic agents [85], whereby their radical scavenging antioxidative activities are from carotenoid and polyphenol groups. The bioactive compounds in mushrooms become raw materials for the development of functional foods. These have the potential to emerge as nutraceutical foods for the next generation [86]. According to Sánchez [87], phenolic compounds, such as myricetin, quercetin, caffeic acid, catechin, and pyrogallol, are present in mushrooms. In addition to these, antioxidant components, such as phenolics, carotenoids, ascorbic acid, tocopherols, ergosterol, and polysaccharides, are also found localized in mushrooms [87].

Various factors impact the antioxidant activity of mushrooms, including the culture and processing conditions in industrial and domestic environments, as well as digestion and absorption in human intestines [88]. Mujić et al. [23] evaluated the phenolics, flavonoids, and the scavenging capacity on DPPH radicals of *Lentinula edodes*, *Hericium erinaceus*, and *Agrocybe aegerita*. They were confirmed to have high total phenolics (23.07 mg GAE/g) and total flavonoid (5.04 mg CE/g) contents. Keleş et al. [89] evaluated the total phenolic and antioxidant activity in 24 dried wild edible mushrooms. The total phenolics levels were highest in *Boletus* and *Leccinum scabrum* showed the highest radical scavenging activity (97.96%). It was found that p-coumaric, p-hydroxybenzoic, protocatechuic and cinnamic acids were extracted from five wild mushrooms [90]. Thus, edible mushrooms may be applied as natural antioxidants in food products. 

## 4. Commercialization of Mushroom and Mushroom Products

Mushrooms have significant health and nutritional benefits and can resolve many problems of under-nutrition and malnutrition. Yet, their cultivation and utilization are still underdeveloped, owing to the fact that mushrooms are highly perishable. It is in these lines that value-added products based on mushrooms are being thought about. These will cater to the protein and micronutrient requirements of the masses and will also troubleshoot issues related to short shelf-life mushrooms, including postharvest losses.

In terms of processing, drying and canning are the two most popular methods of preservation that are applied for the storage of mushrooms. For short-duration preservation, mushrooms are stored in salt solution; this procedure is called steep preservation. Mushrooms enter the arena of food industries in three categories, as either fresh, dried, or canned processed mushroom-based products [91]. Most of the fresh mushrooms are used in regular household/restaurant cooking, and incorporated in soups, sauces, and as fillings in buns or pizzas. Fresh mushrooms are usually sold in local fresh markets because of their short shelf life. Akbarirad et al. [92] and Keles et al. in 2020 [68] reported that the shelf life of mushrooms is limited under normal refrigeration conditions; this becomes a major constraint in the distribution and marketing of fresh products. It is in this direction, to preserve this high-quality and nutritional food type and to ensure that the mushrooms can be used for longer periods, that various mushroom-based products are being developed. Canned mushrooms are very popular and are added to soups, stews, and pizzas instead of fresh mushrooms [93,94,95,96,97,98]. Dried mushrooms are used in instant soup and sauce preparation [99]. The dry form of the mushroom has limited uses compared to powdered mushrooms.

Functional foods are those that are similar to nutritional supplements, therapeutic diets, vita foods, phytochemicals, myo-chemicals and pharma-foods as well, which might be consumed to improve health. Mushrooms fit very well into this category of functional foods, as they are capable of mitigating illnesses. ‘Mushroom nutraceuticals’ include the traditional preparations that become available as health tonics, extracts, concentrates, fermented beverages, tinctures times, teas, soups, herbal formula, powders and arid healthful food dishes [100]. The terminology “mushroom nutraceuticals” was coined by Chang and Buswell [101] and it is now accepted that consistent intake of mushrooms or their products is highly instrumental in averting and treating particular ailments [102] (Chang and Miles 2004).

Several studies report the use of mushrooms as food additives. Süfer et al. [99] mentioned that 5% of *Agaricus bisporus* and *Pleurotus ostreatus* powder in snacks and meatballs contributed towards the production of aromatic and novel foods. The use of mushrooms in meatballs has been well received, owing to the high protein content of mushrooms, as well as the presence of iron, zinc, selenium, potassium, and vitamin B [86,103] and the delicate taste. Consuming excessive red meat results in cardiovascular diseases, cancer, and obesity due to the high levels of saturated fatty acids. The added mushroom powder is expected to reduce the possibility of developing these diseases. Mushrooms are also promoted as mushroom powder; this has a tremendous potential in becoming an ingredient in various food products due to its functional characteristics. Mushroom powder includes dried mushrooms that are pulverized into powder. Many varieties of mushroom powders have been commercialized and can be incorporated into any mushroom dishes. The advantage of using mushroom powders is that a higher concentration of nutrients is obtained in the powdered form. Mushroom powders have been supplemented into noodles, pasta, rice porridge and breads, cakes, muffins and biscuits [104,105,106,107]. Mushrooms are also mainly being used as a composite flour in bakery production. According to Coelho and Salas Mellado [108], nowadays, the substitution of various flour types for wheat flour to satisfy the demands for healthier food attracts significant attention. Higher protein content promotes the development of a better gluten network and produces the right elasticity required for bakery products, especially in pastas and noodles. The additional number of mushrooms in pasta enhance its antioxidant content [16]. Ishara et al. [109] fortified maize flour with mushroom flour from *Agaricus bisporus* and *Pleurotus ostreatus*; a significant increase in the protein content of maize flour with increasing mushroom flour content was evident [109]. The addition of 10% powdered desert truffles is reported to increase the diameter and thickness of the biscuit. The enrichment of protein and increased moisture content in pasta and noodles by adding mushrooms is also documented. Chun et al. used shiitake mushrooms to make pork patties. This powdered mushroom is a source of phosphate in pork patties. Phosphate functions as a food additive, by increasing the water holding capacity, reducing cooking losses and improving the texture of food products. In addition, it also protects the aroma and accelerates the formation of the cured meat color [110]. Mushroom powder seasonings are filled with antioxidants, protein, fiber, amino acids and micronutrients, including B vitamins.

**Figure 3:**
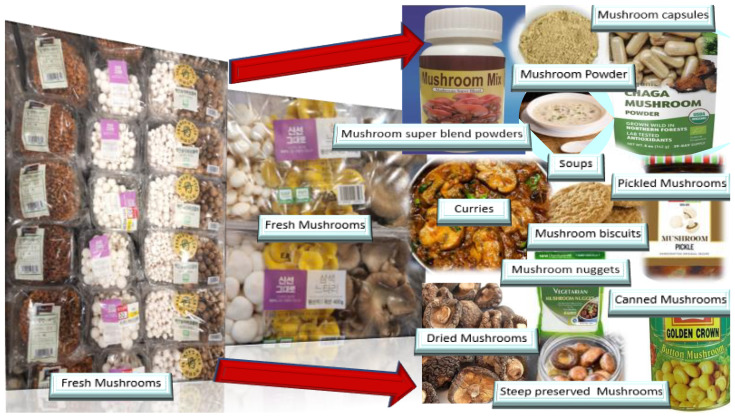
Nutritionally rich mushroom-based foods.

The other product on the market is mushroom capsules, where powdered mushroom is sold in capsular forms. The other product on the market is mushroom powder blends, where various mushrooms with diverse yet unique properties are combined in optimized proportions. The product Super Mushroom Complex: A Formula Of 6 Powerful Mushrooms is a commercialized mushroom blend. This Super Mushroom Complex is an equal mix of the following six mushrooms renowned for their health benefits: lion’s mane, reishi, chaga, shiitake, cordyceps and maitake. Figure 3 gives an overview of the commercialized mushroom products available for consumers.

## 5. Current Knowledge, Future Trends and Recommendations

Mushrooms have many benefits to offer, especially in terms of nutrition, as well as nutraceuticals. However, many obstacles and challenges remain. There is a lot of debate on the unstable nutritional values of mushrooms. It is well known that they are affected by changes in climate, age and many other factors. Harvesting, processing and post-harvest processing impact the nutritional status of mushrooms severely. This is something that cannot be overlooked, since the promised item should be delivered. Even the substrate that is used to grow the mushroom is said to affect the outcome; this is, therefore, a highly sensitive issue. It is, indeed, a challenge to intricately optimize mushrooms; however, it is mandatory. For example, if the output of a mushroom that is expected to have high levels of a bioactive compound has been affected by some external factor, then the end product will, in turn, exhibit reduced bio efficacy. Whether it is mushrooms in their natural state or those processed, dried, powdered, canned, the promised content of the component should be delivered. There is still a long way to go, since most mushroom farming practices are yet to be standardized. An optimized and standardized farming protocol, which dictates the ideal conditions for maximized output in terms of yield, as well as nutritional quality, needs to be unanimously followed to overcome this challenge.

For successful marketing, it is important that a product possesses good, consistent quality with proper details of its nutrition. The concept of total quality management (TQM) should also be applied in commercial mushroom production. This means that the same quality in every batch right from the first packet to the last packet should be maintained. When the mushroom source itself has varying values, then the variance will further expand with the processing conditions and the final mushroom product will be tremendously compromised. This becomes a serious concern when mushroom products are used for sensitive applications (clinical, medical, nutraceutical), since people need to receive what they believe they are promised. In addition, regarding the quality checks, commercialized mushroom products should be cross validated, if they are indeed delivering what they have labelled [111]. When it comes to natural products, the chances of adulteration are high. So, suitable policies and guidelines and governing bodies need to be instituted, and they must closely follow these products. Licensed quality control experts should be also involved in assessing the nutritional values of the generated mushrooms and setting the standards for the quality of commercial mushrooms products, value-added products and processed foods. This will ensure that truly nutritionally rich mushrooms will go into production, and others that fall short of the set standards will not be released for further processing.

The other challenge is that in terms of sensory characteristics, most of the food products supplemented with powdered mushrooms have lesser appeal according to the panelists. The color of the food products supplemented with mushroom powder is darker, and hence were less appealing; this adversely affected the preference of most mushroom-based products [112]. Dr. Yang of Penn State University applied the CRISPR-CAS9 technology to edit the genes of the button mushroom to reduce browning. Such technologies that help overcome the oxidation-based browning of mushrooms will help overcome the color discoloration [113].

Mushrooms, with all their resourcefulness, are yet to hit the consumer headlines, owing to the various challenges facing its marketing aspects. Mushroom varieties are still only available in Chinese, Japanese and Korean countries, whilst other countries only sell 3–4 varieties. In addition to these, there is a whole range of wild mushroom varieties whose nutritional aspects are unexplored. Edible mushrooms may be just the tip of the iceberg when it comes to nutritional aspects. Lack of public awareness, lack of available markets, high transportation cost, large number of middlemen, very limited wholesale market, lack of advertising and storage limitations are a few of the challenges and limiting factors. Somehow, we feel that this age-old tradition of mushroom culture and its applications have not been impacted by the trending technologies. It is strange that when so many obstacles have been overcome through harnessing the available state-of-the-art techniques, not much has been invested into mushrooms. There is undoubtedly room for future development.

## 6. Concluding Remarks

Food-based applications, as well as the nutritional aspects of mushrooms, were reviewed. The various mushrooms available in the Asian markets and their nutritional value and importance as nutrient-rich foods have been discussed. The varied vitamins, minerals, proteins, fats, carbohydrates available in these natural reservoirs have been projected. The commercialized mushroom-based value-added products have been presented, and the challenges confronting their widespread utility, despite their high nutritional contents, have been discussed. The need to improvise and the obstacles that must be overcome have also been addressed.

## Figures and Tables

**Table 1:** Nutritional aspects of edible mushrooms.

Edible Mushrooms	Common Name	Proteins (g/100 g DW)	AA (g/100 g DW)	Ash (g/100 g DW)	CH (g/100 g DW)	Fats (g/100 g DW)	Fibers (g/100 g DW)	Sugars (g/100 g DW)	Vitamins (Units/100 g DW)	Minerals (mg/Kg DW)	Reference
*Agaricus arvensis*	Horse mushroom	32.87	ND	0.18	32.91	ND	0.14	ND	ND	ND	[54]
*Agaricus arvensis*	Horse mushroom	ND	ND	ND	ND	ND	ND	ND	ND	Ca (1090–1280), K (43,432), Mg (890–1070), Na (230 N-83), P (13,419), B (3.35), Cu (45–187), Fe (740–1056), Mn (33–87.5)	[55]
*Agrocybe aegerita*	Pioppino mushroom	6.68		6.69	84.5	2.13		13.42	ND	ND	[56]
*Agrocybe aegerita*	Pioppino mushroom	2.05 ^†^	1.42 ^†^	087 ^†^	6.07 ^†^	0.51 ^†^	ND	Glucose (0.0203) ^†^, fructose (0.0114) ^†^	Ascorbic acid (0.0082) ^†^	NH_4_ (0.196), Ca (0.098), Mg (0.563) ^†^, K (3.359) ^†^, Na (0.004) ^†^ g/kg	[57]
*Agaricus bisporus*	Brown button mushroom	1.29 ^†^		0.95 ^†^	5.98 ^†^	0.14 ^†^		4.27 ^†^	ND	ND	[58]
*Agaricus bisporus*	Button mushroom	26.7%	ND	11.5%	57.2%	4.6%	ND	ND	B_1_ (1.24), B_2_ (5.06), B_3_ (362), ascorbic acid (80) mg/100 g DM. Pyridoxine (3), pyridoxal (12), pyridoxamine (937) µg	ND	[59]
*Agaricus bisporus*	Wild/button mushroom	ND	ND	ND	ND	ND	ND	ND	ND	Ca (860–1400), K (38,105–40,371), Mg (1099–1400), Na (545–957), P (10,430–12,475), B (3.7–19.4), Cu (3–65), Fe (44–190), Mn (5.7–28.8) mg/kg	[55]
*Agaricus bisporus*	Wild button mushroom	29.5	ND	10.0%	2.1%	0.1%	1.23%	ND	ND	K (15.5), Ca (7.5), Mg (6.3), Zn (1.5), Mn (0.4), Ni (0.2)%	[60]
*Agaricus bisporus*	Brown button mushroom	33.61–53.7	0.028–0.051	7.01–13.26	ND	0.85–1.74	4.99–8.41	Fructose (0.33–3.58), glucose (2.17–6.59), mannose (0.02–0.1)	ND	ND	[61]
*Agaricus bisporus*	Brown button mushroom	17.25	ND	13.84	66.33	2.58	11.58	ND	ND	ND	[62]
*Agaricus bisporus*	White button mushroom	ND	ND	ND	ND	1515.7	ND	ND	ND	ND	[63]
*Agaricus bisporus*	White button mushroom	1.23 ^†^	ND	0.85 ^†^	6.46 ^†^	0.19 ^†^		5.79 ^†^	ND	ND	[58]
*Agaricus bisporus*	Wild button mushroom	30.44	Essential amino acids (34.64) and non essential amino acids (31.91)	9.25	ND	ND	ND	ND	Ascorbic acid (920 µg)	K (27,940), Ca (2505), Na (1988), Mg (1450), Fe (554), P (452), Zn (139), Cu (68), Mn (4.32), Se (0.62) mg/kg)	[64]
*Agaricus bisporus*	White button mushroom	18.53	ND	12.68	66.75	2.04	10.28	ND	ND	ND	[62]
*Agaricus bisporus*	Button mushroom	29.5–37.6	4.51–8.89	ND	ND	0.0056–0.0174	ND	ND	ND	ND	[65]
*Agaricus bitorquis*	Pavement mushroom	23.2	ND	8.56%	1.51%	0.08%	1.33%	ND	ND	K (15.5), Ca (5.9), Mg (7.3), Zn (1.8), Mn (0.5), Cr (0.01), Cd (0.01), Ni (0.4)%	[60]
*Agaricus campestris*	Field mushroom	25.7	ND	11.4%	1.0%	0.04%	1.45%	ND	ND	K (16.3), Ca (6.0), Mg (5.5), Zn (1.2), Mn (0.6), Cr (0.05), Ni (0.3)%	[60]
*Agaricus campestris*	Field mushroom	0.0506–0.0555	Proline (147.1–149.61 µmol/g)	ND	5.062–7.489	ND	ND	Glucose (20.25–29.96), sucrose (24.55–34.56)	ND	ND	[66]
*Agaricus campestris*	Field mushroom			16.7						K (44.4), P (1.46), Ca (380), Na (114), Fe (752), Cu (452) mg/kg	[67]
*Agaricus* *langei*	The great wood mushroom	35.14	ND	14.1	34.83	ND	3.28	ND	ND	ND	[54]
*Agaricus* *langei*	The great wood mushroom									K (5386), Mg (1273), Ca (238), Mn (13.98), Fe (104.18), Zn (47.19), Cu (46.85) mg/kg	[68]
*Agrocybe cylindracea*	Chestnut mushroom	19.65	ND	8.75	70.95	1.05	7.94	ND	ND	ND	[62]
*Amanita vaginata*	The grisette	ND	ND	ND	ND	ND	ND	ND	ND	Cu (60.25), Mn (69.9), Zn (104.61), Ni (15.29), Fe (1631.86), Al (3349.02) mg/kg	[69]
*Armillaria mellea*	Honey fungus	ND	1.34–10.7%	ND	ND	ND	ND	48.93–62.9%	Ascorbic acid (2.55–2.99 mg AAE)	ND	[70]
*Auricularia auricula-judae*	Jelly ear	36.3	ND	7.07	33.23	ND	2.81	ND	ND	ND	[54]
*Auricularia auricula-judae*	Jelly ear	6.49–11.71	5.10–9.75%	3.56–5.61%	ND	0.50–0.81%	6.10–9.20%	ND	ND	ND	[71]
*Auricularia auricula-judae*	Jelly ear	23.75	ND	10.4%	38.3%	6.6%	6.45%	ND	ND	ND	[72]
*Auricularia polytricha*	Wood ear	ND	ND	ND	ND	531.3	ND	ND	ND	ND	[63]
*Boletus aestivalis*	Summer Bolete	32.76	ND	14.97	52.07	ND	12.13	ND	ND	ND	[54]
*Boletus loyo*	Chilean porcini	21.25	ND	7.56	67.94	3.25	15.65	ND	ND	ND	[62]
*Cantharellus cibarius*	Girolle	34.17	ND	7.78	47.0	ND	1.4	ND	ND	ND	[54]
*Cantharellus cibarius*	Girolle	4.75–4.78	Proline (114–122.49 µmol/g)	ND	4.653–6.692	ND	ND	Glucose (18.61–26.77) sucrose (34.93–38.41) µg/g DW	ND	ND	[66]
*Cantharellus cibarius*	Girolle	ND	ND	ND	ND	ND	ND	ND	ND	K (15,747), Mg (686), Ca (439), Mn (18.73), Fe (174.42), Zn (82.22), Cu (2.91) mg/kg DW	[68]
*Cantharellus cibarius*	Girolle	ND	ND	ND	ND	ND	ND	ND	ND	Ca (47.2), Mg (572), K (39,600), Na (181), P (4690), B (4.03), Cu (34.8), Fe (142), Mn (62.2), Zn (108)	[73]
*Clavariadelphus pistillaris*	Giant club	16.27	ND	20.77	62.37	0.59	ND	ND	ND	ND	[74]
*Clitopilus prunulus*	The miller	18.13	ND	30.19	50.66	1.01	ND	ND	ND	ND	[75]
*Coprinus comatus*	Shaggy ink cap	15.67	ND	12.85	70.36	1.13	ND	ND	ND	ND	[76]
*Coprinus comatus*	Shaggy ink cap	23.07	ND	13.24	40.42	2.04	21.13	ND	ND	Pb (0.172), Cd (0.14), Hg (0.019), As (0.38), Fe (1471), Mg (1334), Cu (10.17), Zn (31.73), Se (0.51) mg/kg DW	[77]
*Coprinus comatus*	Shaggy ink cap	ND	ND	14.6%	ND	ND	ND	ND	ND	K (35.4), P (8.64), Ca (2840), Na (547), Fe (584), Cu (74.8)	[67]
*Cordyceps militaris*	Scarlet caterpillar club	ND	ND	ND	ND	ND	ND	ND	ND	As (0.0047), Pb (0.22), Cd (0.0041), Cu (9.1), Ag (0.024), Zn (5.2), Mn (1.6)	[78]
*Cordyceps militaris*	Scarlet caterpillar club	33.44	ND	0.49	53.31	2.3	6.02	ND	ND	ND	[79]
*Cortinarius lebre*	Lebre (Chilean name)	23.88	ND	8.8	66.32	1.0	11.92	ND	ND	ND	[62]
*Cyttaria espinosae*	Digüẽne (Chilean name)	17.46	ND	4.9	71.55	6.09	8.05	ND	ND	ND	[62]
*Flammulina velutipes*	Golden needle mushroom	0.47	ND	0.88 ^†^	10.57 ^†^	0.21 ^†^	ND	8.29 ^†^	ND	ND	[58]
*Flammulina velutipes*	Golden needle mushroom	17.89	ND	9.42	70.85	1.84	10.36	ND	ND	ND	[62]
*Grifola gargal*	Gargal (Chilean name)	9.9	ND	5.31	82.6	2.19	14.09	ND	ND	ND	[62]
*Hericium erinaceus*	Lion’s mane	ND	ND	ND	ND	1.599	ND	ND	ND	ND	[63]
*Hypsizigus marmoreus*	Beech mushroom	ND	ND	ND	ND	1.245–1.738	ND	ND	ND	ND	[63]
*Hypsizygus tessulatus*	Beech mushroom	37.8	ND	9.09	51.2	ND	12.9	ND	ND	ND	[54]
*Lactarius deliciosus*	Red pine mushroom	ND	ND	7.16%	ND	ND	ND	ND	ND	K (25.4), P (5.07), Ca (430), Na (86.3), Fe (1190), Cu (8.05) mg/kg DM	[67]
*Lactarius deliciosus*	Red pine mushroom	ND	ND	ND	ND	ND	ND	ND	ND	K (7121), Mg (579), Ca (222), Mn (12.45), Fe (144.01), Zn (57.12), Cu (8.85)	[67]
*Lactarius deliciosus*	Red pinemushroom	18.02	ND	11.04	67.36	3.58	10.45	ND	ND	ND	[62]
*Lactarius hygrophoroides*	Hygrophorus milky	44.93	ND	2.0	42.0	ND	10.58	ND	ND	ND	[54]
*Leccinum aurantiacum*	Red-capped scaber stalk	ND	ND	ND	ND	ND	ND	ND	ND	Cu (41.11), Mn (19.3), Zn (77.33), Ni (9.1), Fe (227.38), Al (480.28)	[69]
*Lentinula edodes*	Shiitake	ND	ND	8.42%	ND	ND	ND	ND	ND	K (31.6), P (8.8), Mg (1.46), Ca (83.3), Na (119), Fe (36.9), Cu (12.2), Zn (74.6)	[67]
*Lentinus edodes*	Shiitake	16.14	ND	6.74	74.34	1.78	15.24	ND	ND	ND	[62]
*Lentinus edodes*	Shiitake	ND	Alanine (0.853) ^†^, valine (0.371) ^†^, and isoleucine (0.619) ^†^	ND	ND	ND	ND	ND	ND	ND	[80]
*Lentinula edodes*	Shiitake	ND	ND	ND	ND	1.244–1.388	ND	ND	ND	ND	[63]
*Lentinula edodes*	Shiitake	0.89 ^†^	ND	1.36 ^†^	17.62 ^†^	0.35 ^†^	ND	14.08 ^†^	ND	ND	[58]
*Lentinula edodes*	Shiitake	21.24–29.15	11,778–19,792	4.06–5.92	ND	0.75–1.02	ND	Arabitol (3.15–7.78), fructose (0.12–1.51), mannitol (5.46–8.3), glucose (0.81–1.2), trehalose (5.37–9.6)	ND	ND	[81]
*Lepiota magnispora*	Yellow foot dapperling	27.55	ND	3.05	35.0	ND	5.2	ND	ND	ND	[54]
*Lepista irina*	Flowery blewit	26.12	ND	3.16	50.2	ND	6.08	ND	ND	ND	[54]
*Macrolepiota procera*	Parasol mushroom	ND	ND	11.6%	ND	ND	ND	ND	ND	K (33.1), P (11.9), Ca (1340), Na (124), Fe (617), Cu (83.8).	[67]
*Macrolepiota procera*	Parasol mushroom	8.56	ND	5.69	83.65	2.1	16.31	ND	ND	ND	[62]
*Morchella conica*	Morel	ND	ND	15.0%	ND	ND	ND	ND	ND	K (25.9), P (14.6), Mg (6.71), Ca (12,900), Na (86.7), Fe (1110), Cu (128), Zn (195)	[67]
*Morchella conica*	Morel	20.56	ND	9.87	68.05	1.52	21.63	ND	ND	ND	[62]
*Panus fulvus*	Trumpet-like mushroom	27.06	ND	3.11	33.04	ND	6.08	ND	ND	ND	[54]
*Pleurotus ostreatus*	Oyster mushroom	8.68	ND	9.33	70.03	1.34	11.1	ND	ND	ND	[82]
*Pleurotus ostreatus*	Oyster mushroom	ND	ND	ND	ND	0.933–1.791	ND	ND	ND	ND	[63]
*Pleurotus ostreatus*	Oyster mushroom	18.35	ND	7.82	71.25	2.58	14.31	ND	ND	ND	[62]
*Pleurotus ostreatus*	Oyster mushroom	0.76 ^†^	ND	0.62 ^†^	9.30 ^†^	0.15 ^†^	ND	4.97 ^†^	ND	ND	[58]
*Pleurotus eryngii*	King oyster mushroom	ND	ND	ND	ND	1.215	ND	ND	ND	ND	[63]
*Pleurotus eryngii*	King oyster mushroom	1.21 ^†^	ND	0.68 ^†^	8.95 ^†^	0.16 ^†^	ND	8.67 ^†^	ND	ND	[58]
*Pleurotus eryngii*	King oyster mushroom	22.1	Essential aminoacids (2.631) and non--essential amino acids (3.279)	6.5	63.4	2.5	ND	2.9	ND	ND	[83]
*Pleurotus eryngii*	King oyster mushroom	ND	ND	8.77%	ND	ND	ND	ND	ND	K (25.3), P (9.19), Mg (2.07), Ca (240), Na (579), Fe (189), Cu (11.2), Zn (57.6)	[67]
*Pleurotus eryngii*	King oyster mushroom	ND	ND	ND	ND	ND	ND	ND	ND	K (7839), Mg (1838), Ca (205), Mn (8.15), Fe (103.86), Zn (56.69), Cu (9.39)	[68]
*Pleurotus pulmonarius*	Santali	37.63	ND	10.17	43.4	ND	4.12	ND	ND	ND	[54]
*Pleurotus pulmonarius*	Santali	37.63	ND	ND	43.4%	1.93%	4.12%	ND	ND	ND	[84]
*Pleurotus pulmonarius*	Santali	7.88	ND	9.31	60.8	1.7	11.54	ND	ND	ND	[82]
*Pleurotus pulmonarius*	Santali	ND	ND	ND	ND	1.719	ND	ND	ND	ND	[63]
*Pleurotus tuberregium*	King tuber mushroom	3.53	ND	1.27	85.81	0.11	9.29	ND	ND	Ca (1.283), Mg (1.121), K 3.743), Mn (0.003), Cu (0.0018), Zn (0.0047), Fe (0.027)	[82]
*Ramaria flava*	Changle (Chilean name)	16.92	ND	8.83	72.1	2.15	11.81	ND	ND	ND	[62]
*Ramaria botrytis*	Changle (Chilean name)	16.85	ND	7.91	74.0	1.24	9.98	ND	ND	ND	[62]
*Ramaria subaurantiaca*	Changle (Chilean name)	15.87	ND	8.25	74.0	1.88	10.3	ND	ND	ND	[62]
*Sarcodon imbricatus*	Scaly hedgehog	ND	ND	ND	ND	ND	ND	ND	ND	Cu (66.16), Mn (7.18), Zn (112.29), Ni (5.38), Fe (35.45), Al (94.49)	[69]
*Schizophyllum commune*	Split gill	22.5	ND	10.1	32.43	ND	6.5	ND	ND	ND	[54]
*Suillus bovinus*	Jersey cow mushroom	14.25	ND	11.24	71.23	3.28	11.23	ND	ND	ND	[62]
*Suillus granulatus*	Weeping bolete	16.54	ND	12.25	67.7	3.51	12.58	ND	ND	ND	[62]
*Suillus lakei*	Matte Jack	17.9	ND	13.35	64.71	4.04	12.74	ND	ND	ND	[62]
*Suillus luteus*	Slippery Jack	ND	ND	8.08%	ND	ND	ND	ND	ND	K (19.2), P (5.3), Mg (1.22), Ca (260), Na (175), Fe (819), Cu (8.16), Zn (51.1)	[67]
*Suillus luteus*	Slippery Jack	ND	ND	ND	ND	ND	ND	ND	ND	K (6449), Mg (736), Ca (184), Mn (7.53), Fe (113.96), Zn (52.3), Cu (14.84)	[67]
*Suillus luteus*	Slippery Jack	13.58	ND	6.74	76.23	3.45	11.85	ND	ND	ND	[62]
*Tricholoma terreum*	Grey knight	18.72	ND	13.49	65.93	1.86	12.3	ND	ND	ND	[62]
*Xerocomus chrysenteron*	Red cracking bolete	20.5	ND	13.69	62.97	2.84	8.33	ND	ND	ND	[62]

DW—Dry weight; ^†^—wet weight; AA—amino acids; CH—carbohydrates.

## Data Availability

Not applicable.
